# Transcriptomic and Functional Analyses of Two Cadmium Hyper-Enriched Duckweed Strains Reveal Putative Cadmium Tolerance Mechanisms

**DOI:** 10.3390/ijms241512157

**Published:** 2023-07-29

**Authors:** Gui-Li Yang, Lei Huang, Xiao Yang, Zhu Li, Hai-Min Liao, Kang Mao, Zhao-Ju Liu, He-Yan Geng, Qin Cao, Ai-Juan Tan

**Affiliations:** 1 Key Laboratory of Plant Resource Conservation and Germplasm Innovation in Mountainous Region (Ministry of Education), Collaborative Innovation Center for Mountain Ecology & Agro-Bioengineering (CICMEAB), College of Life Sciences/Institute of Agro-Bioengineering, Guizhou University, Guiyang 550025, China; glyang3@gzu.edu.cn (G.-L.Y.); yangxiao98103@163.com (X.Y.); zhuliluck@163.com (Z.L.); lhaimin@163.com (H.-M.L.); lzhju6@163.com (Z.-J.L.); hygeng2022@163.com (H.-Y.G.); caoqin9909@163.com (Q.C.); 2 Institute of Geochemistry, Chinese Academy of Sciences, Guiyang 550081, China; maokang@mail.gyig.ac.cn; 3 The Key Laboratory of Chemistry for Natural Products of Guizhou Province and Chinese Academy of Sciences, Guiyang 550014, China; xinyanghuanglei@163.com

**Keywords:** cadmium, duckweed, heavy metals, hyperaccumulator, transcriptome

## Abstract

Cadmium (Cd) is one of the most toxic metals in the environment and exerts deleterious effects on plant growth and production. Duckweed has been reported as a promising candidate for Cd phytoremediation. In this study, the growth, Cd enrichment, and antioxidant enzyme activity of duckweed were investigated. We found that both high-Cd-tolerance duckweed (HCD) and low-Cd-tolerance duckweed (LCD) strains exposed to Cd were hyper-enriched with Cd. To further explore the underlying molecular mechanisms, a genome-wide transcriptome analysis was performed. The results showed that the growth rate, chlorophyll content, and antioxidant enzyme activities of duckweed were significantly affected by Cd stress and differed between the two strains. In the genome-wide transcriptome analysis, the RNA-seq library generated 544,347,670 clean reads, and 1608 and 2045 differentially expressed genes were identified between HCD and LCD, respectively. The antioxidant system was significantly expressed during ribosomal biosynthesis in HCD but not in LCD. Fatty acid metabolism and ethanol production were significantly increased in LCD. Alpha-linolenic acid metabolism likely plays an important role in Cd detoxification in duckweed. These findings contribute to the understanding of Cd tolerance mechanisms in hyperaccumulator plants and lay the foundation for future phytoremediation studies.

## 1. Introduction

Cadmium (Cd) is a highly toxic metal that is widely found in the environment, and soil contamination with Cd has been reported worldwide [1]. In addition, heavy metal accumulation can reduce organismal productivity at toxic levels [2,3,4,5]. For example, Cd inhibits plant growth and development by binding to amino acids containing sulfhydryl groups and interfering with plant environmental homeostasis [6]. Compared with many other heavy metal compounds, Cd compounds are more soluble. Thus, Cd is easily absorbed and adsorbed by plants. It frequently accumulates in various plant parts, through which it enters the food chain [7,8]. It is easily enriched as it moves up the food chain, thus jeopardising higher levels of the biota [9,10,11]. Therefore, Cd contamination not only affects plant yield and quality but also poses a significant threat to human health through the food chain [12]. Humans who consume excessive Cd are at risk of developing conditions such as immune suppression, obstructive lung disease, emphysema, and permanent kidney failure [13]. Furthermore, Cd can potentially cause lung, bladder, kidney, prostate, and breast cancers and has been classified as a carcinogen by the International Agency for Research on Cancer (IARC) [13,14,15]. Therefore, there is an urgent need to elucidate the mechanisms underlying Cd tolerance in plants and to remediate Cd pollution.

Cd affects plant growth and development by influencing many critical physiological processes, such as inducing cell death, damaging genomic DNA, impairing photosynthesis, disrupting intracellular homeostasis, inducing oxidative stress, and initiating lipid peroxidation [16,17,18,19,20,21]. Plants have developed various detoxifying methods, including root retention, compartmentalisation, chelation, phytonutrient production, and antioxidant properties, to reduce the negative effects of Cd [22,23,24]. For instance, it has been discovered that root secretions and root cell walls interact with Cd to produce complexes or precipitates, which localizes Cd on the root epidermis or in the root cell walls and prevent it from injuring plant cells by further penetrating plant stems and leaves [25,26,27]. Zheng et al. [28] found that as the time of Cadmium stress increased, duckweed distributed Cd in a less toxic HCl-extracted state and HAc-extracted state, mostly by retention in the root cell wall and sequestration in the leaf vacuoles, and is dynamically modified. Small molecules, such as phytochelatin (PC), metallothionein (MT), organic acids, and amino acids, form chelates with Cd^2+^ in the cytoplasmic matrix. Chmur et al. [29] found that PC levels were increased by Cd treatment in response to Cd toxicity. They are then transported to the vacuole by transport proteins, which reduces their toxicity to the remainder of the plant [30,31,32,33,34]. In duckweed, it was observed that roots (2.05–95.52%) accumulated more Cd than leaves (0.14–16.98%) in the early stages of Cd stress and that Cd was transferred from roots (6.79~66.23%) to leaves (46.64~92.83%) with time [28]. Additionally, the translocation of Cd^2+^ from the cytoplasm to the vesicles reduced damage by Cd^2+^ to proteins in the cytoplasm. For example, Krotz et al. [35] found that the concentration of Cd^2+^ accumulated in vesicles was approximately 38 times higher than that in the cytoplasm [35]. Nutrients such as Ca, Fe, and Zn can improve the tolerance of plants to Cd [36]. Vidaković-Cifrek et al. [37] found that the addition of Zn increased the pigment content and restored the activity of photosystem II (PS II) in Cd-stressed *Lemna minor* L. and increased the tolerance of *Lemna minor* L. to Cd. The Ca^2+^ signal response in *Lemna turionifera* 5511 during Cd stress has been found [38]. In addition, because Ca^2+^ and Cd^2+^ compete for the same Ca^2+^ channels in plants, Ca^2+^ as a plant nutrient helps reduce Cd^2+^ toxicity [3]. The alleviation of cadmium toxicity in *Lemna minor* is performed by exogenous salicylic acid [39]. Plants respond to Cd^2+^ stress by increasing antioxidant enzyme activity, which helps maintain metabolic homeostasis. For example, the POD activity of duckweed increased under Cd stress to counteract cadmium toxicity [40]. Plants also respond to Cd stress by up-regulating the expression of SoPCS [41]. Although several studies on the physiological and biochemical phenomena connected to Cd-tolerant plants and related genes have been published, the intricately controlled molecular regulatory system of Cd tolerance in plants remains unclear.

Recently, RNA sequencing (RNA-seq) has been widely used to illustrate the response of plants to biotic and abiotic stresses and to reveal their molecular mechanisms. Many studies have used this sequencing technology to elucidate the response of plants to Cd stress and reveal its molecular mechanisms. Xu et al. [42] found that Cd-tolerant *Landoltia punctata* produced a large number of differentially expressed genes under Cd stress, and that up-regulated genes involved in sulphur and ROS metabolism may cause a high Cd tolerance. Vacuolar sequestration is likely to play an important role in Cd detoxification in *Landoltia punctata*. The expression of Orphans, P2/EREBP-related genes, was significantly higher in Cd-tolerant duckweed than in Cd-sensitive duckweed [43]. In addition, studies have shown that genes related to DNA repair have been up-regulated with riluzole (RIL) treatment under Cd stress [44]. Duckweed is the smallest known monocotyledonous flowering plant. It is often found floating in still water and is widely distributed globally [45]. In addition, duckweed has the characteristics of a fast growth, easy recovery and collection, simple structure consisting only of leaves and roots (which limits long-distance migration of heavy metal ions), and ability to hyperaccumulate Cd. Considering these advantages, it is recognised as a promising ideal species for Cd phytoremediation [46]. Zheng et al. [46] found that Cd enrichment in duckweed (*Lemna minor*) treated with 10 mg/L of Cd^2+^ for 7 d and Cd removal from the water column reached 2834.30 mg/kg and 82.50%, respectively. Although many physiological and biochemical studies have been conducted, the molecular mechanisms underlying Cd tolerance in Cd-hyperaccumulated duckweed remain largely unknown. Therefore, there is an urgent need for genome-wide studies on duckweed.

In this study, two duckweed strains with a Cd-hyper-enrichment capacity were used as experimental materials. Experiments on its growth indexes, antioxidant enzyme activities, and cadmium-accumulation-related indexes revealed significant genotypic differences between high-Cd-tolerance duckweed (HCD) and low-Cd-tolerance duckweed (LCD) in response to cadmium stress in terms of growth and development, photosynthesis, and antioxidant enzyme activity. Therefore, we hypothesised that these two genotypes would differ significantly in their genome-wide responses to Cd stress. In this study, we used bioinformatic tools and methods to analyse the duckweed transcriptome. Samples were treated with 0.5 mg/L of CdCl_2_ for 12 h and used for high-throughput RNA-seq together with control samples. A comparative transcriptome analysis revealed tens of thousands of single-gene expression patterns and helped investigate genome-wide regulation in response to Cd stress. These results offer a theoretical framework for understanding the mechanism of Cd tolerance in duckweed as well as new information for discovering novel genes and genetically enhancing plant resistance to Cd stress.

## 2. Results

### 2.1. Effect of Cd Treatment on Duckweed Growth Indicators

**Figure 1:**
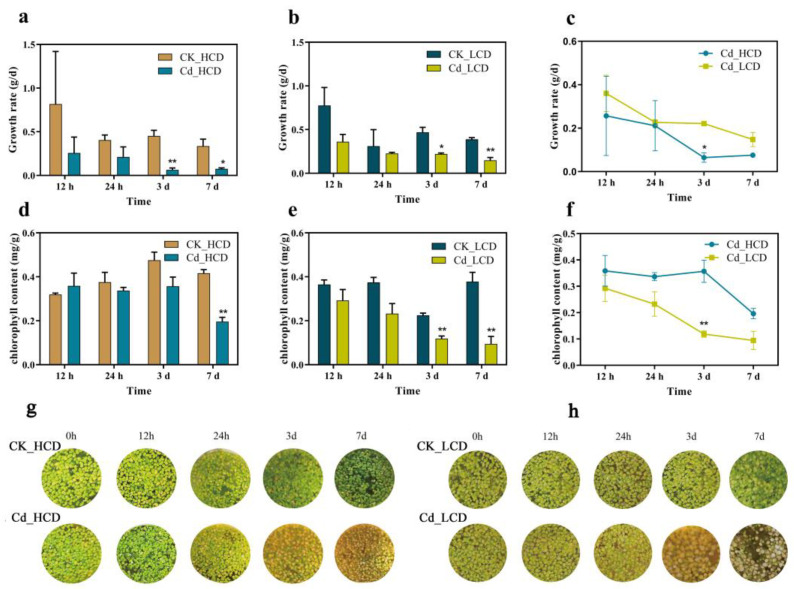
Growth indicators of duckweed under cadmium (Cd) stress at different time points. (**a**) Growth rate of low-Cd-tolerance duckweed (HCD), (**b**) growth rate of high-Cd-tolerance duckweed (LCD), (**c**) comparison of growth rates of HCD and LCD, (**d**) changes in chlorophyll content of HCD, (**e**) changes in chlorophyll content of LCD, (**f**) comparison of chlorophyll content of HCD and LCD, (**g**) growth of HCD, (**h**) growth of HCD under Cd stress. Bars indicate mean ± SE (n = 3). Asterisks indicate significant differences between Cd-treated and control samples at each time point (*: *p* < 0.05, **: *p* < 0.01).

To compare visible differences between HCD and LCD under Cd stress, we observed the growth of the two varieties at 12 h, 24 h, 3 d, and 7 d after the initiation of Cd treatment. The biomass of both HCD and LCD decreased, most notably by 3 and 7 d, and significant decreases in the growth rate were observed (Figure 1a,b). The differences in growth rates between the control and Cd-treated groups after 12 and 24 h of Cd stress were not significant, indicating that the effects of Cd toxicity on duckweed require a certain response time to manifest the phenotype. The growth rate of LCD under Cd stress was significantly higher than that of HCD by 3 d (Figure 1c). This result indicates that there is a great intra-species variation in Cd tolerance in duckweed and that Cd tolerance varies greatly among strains.

After treatment, the chlorophyll content of duckweed in the Cd-treated group decreased to some extent compared to that in the control group. Leaves gradually turned yellow as Cd treatment time increased (Figure 1g,h). In HCD, chlorophyll content decreased significantly by 52.87% (0.20 mg/g) after 7 d (Figure 1d). In LCD, chlorophyll content significantly decreased after 3 d (Figure 1e). The results showed that chlorophyll content gradually decreased with an increasing Cd treatment time. The chlorophyll content of HCD was higher overall than that of LCD at different times of Cd stress and was significantly higher than that of the LCD strain at 3 d, up to 0.24 mg/g (Figure 1f).

### 2.2. Effect of Cd Stress on Cd Concentration and Removal Efficiency of Duckweed

**Figure 2:**
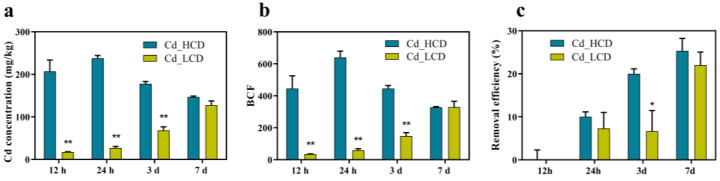
Changes in Cd-related indicators of duckweed at different times of Cd stress. (**a**) Changes in Cd concentration, (**b**) changes in BCF, (**c**) changes in removal efficiency under Cd stress for HCD and LCD. Bars indicate mean ± SE (n = 3). Asterisks indicate significant differences between Cd-treated and control samples at each time point (*: *p* < 0.05, **: *p* < 0.01).

To further investigate the Cd tolerance characteristics of HCD and LCD, Cd concentration, bioconcentration factors (BCFs), and Cd removal efficiency were determined (Figure 2). The Cd concentration of HCD gradually decreased with the increase in Cd treatment time and reached the peak (238.06 mg/kg (DW)) at 24 h. In contrast, the Cd concentration of LCD gradually increased with increasing Cd treatment time and reached a peak (127.63 mg/kg (DW)) at 7 d (Figure 2a). BCF is an important indicator used to measure the ability of plants to enrich pollutants. The BCF of HCD peaked (640.45) at 24 h and gradually decreased with increasing Cd treatment time. The BCF of LCD gradually increased with increasing Cd treatment time from 12 h and peaked at 7 d (329.78) (Figure 2b). The results showed that the BCF of HCD and LCD varied after Cd treatment. The removal efficiency of different duckweed strains differed. The HCD strain exhibited a higher removal efficiency and was significantly higher than that of the LCD strain. The Cd removal efficiency of both duckweed strains increased with treatment time (Figure 2c). The results showed that the removal efficiency of strain HCD was greater than that of strain LCD, and that the effect of Cd concentration could be increased by extending the time when used for environmental remediation.

### 2.3. Effect of Cd Stress on Antioxidant Enzymes and Glutathione Sulfhydryltransferase of Duckweed

**Figure 3:**
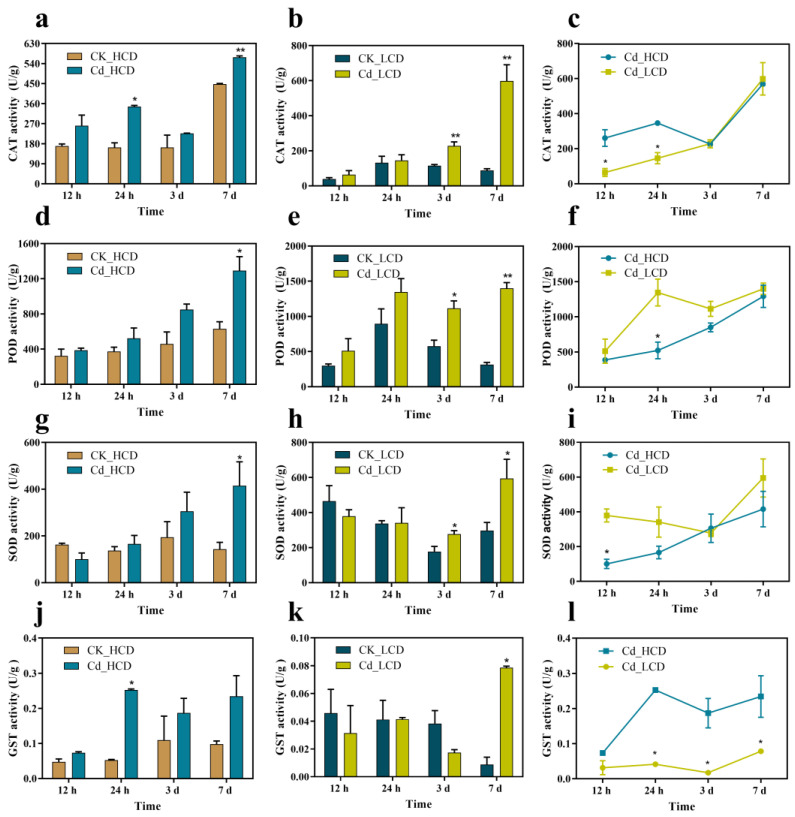
Changes of antioxidant enzymes and glutathione sulfotransferase in duckweed at different times of Cd stress. (**a**) CAT activity of HCD, (**b**) CAT activity of LCD, (**c**) comparison of CAT activity of HCD and LCD, (**d**) POD activity of HCD, (**e**) POD activity of LCD, (**f**) comparison of POD activity of HCD and LCD, (**g**) SOD activity of HCD, (**h**) SOD activity of LCD, (**i**) comparison of SOD activity of HCD and LCD, (**j**) GST activity of HCD, (**k**) GST activity of LCD, (**l**) comparison of GST activity of HCD and LCD under Cd treatment. Bars indicate mean ± SE (n = 3). U/g, one enzyme activity unit, refers to 1 g of tissue that can convert 1 μmol of substrate within 1 min at the optimal temperature. Asterisks indicate significant differences between Cd-treated and control samples at each time point (*: *p* < 0.05, **: *p* < 0.01).

Figure 3a–i illustrates the antioxidant enzyme activities of the two duckweed strains. POD, CAT, and SOD activities of the two duckweed strains gradually increased with increasing Cd stress duration. After 7 d of treatment, POD, CAT, and SOD activities were significantly higher than those of the control (Figure 3a,b,d,e,g,h). The POD activity of the LCD strain was higher than that of the HCD strain (Figure 3f). This may suggest that LCD needs to produce more POD in response to Cd stress. Glutathione S-transferase (GST) is the main system for exogenous detoxification and cellular resistance to damage in plants [47,48,49]. The overall increase in GST activity of both duckweeds was observed with the addition of a 0.5 mg/L Cd treatment compared to the control (Figure 3j,k). The peak GST activity of the HCD strain was 0.2528 U/g at 24 h of Cd stress (Figure 3j). LCD reached its highest activity (0.0786 U/g) at 7 d under Cd stress, which was only one-fourth of the highest value of the HCD strain. The GST activity of the HCD strain was higher than that of the LCD strain at different time points (Figure 3k).

### 2.4. Overview of the Transcriptome of Different Treatment Groups under Cd Treatment

**Figure 4:**
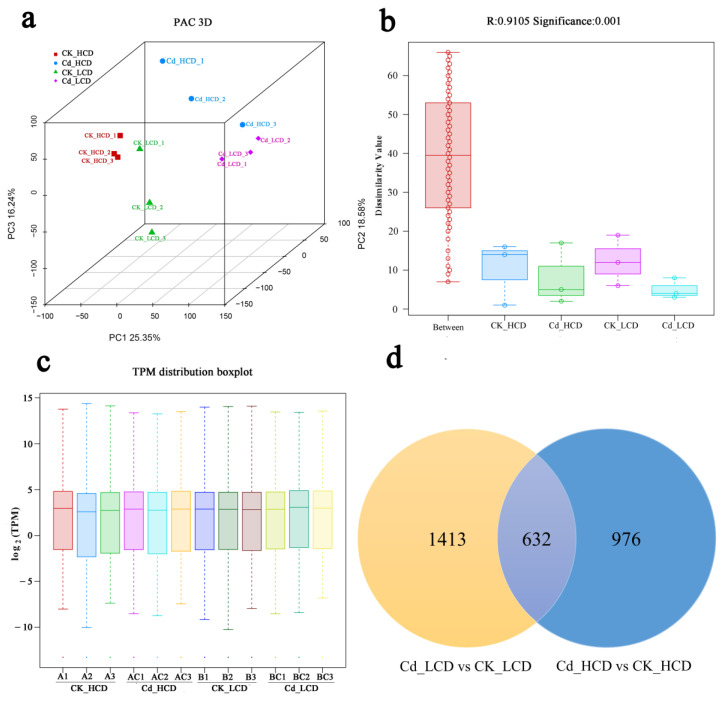
Transcriptomic profiling of duckweed under Cd stress. (**a**) PCA principal component analysis diagram. Points of different colours or shapes represent sample groups under different environments or conditions. The scale of horizontal and vertical coordinates is relative distance and has no practical significance. (**b**) Anosim inter-group similarity analysis box plot. The closer the R-value is to 1, the greater the difference between groups; the closer the R-value is to 0, the less significant the difference between and within groups. *p* < 0.001 indicates statistical significance. (**c**) Box plot of gene expression. The box plot of each region has five statistics (maximum, upper quartile, median, lower quartile, and minimum, respectively, from top to bottom). The TPM (Transcripts Per Kilobase of exon model per Million mapped reads) is obtained by normalising the raw count data to ensure comparability between samples. Different colours represent different samples. (**d**) Venn diagram of log_2_ differential genes.

To further understand the molecular mechanisms of Cd tolerance in duckweed, samples from two duckweed strains exposed to 0.5 mg/L of CdCl_2_ for 12 h were subjected to a transcriptome analysis. After raw data filtering, a total of 544,347,670 clean reads were generated from all sample libraries. There were approximately 41,233,077–54,521,819 clean reads from each sample, with an average GC content of 54.18–55.00%. Additionally, the Q20 and Q30 values for HCD and LCD exceeded 97.26% and 89.59%, respectively. The rate of clean read mapping to the reference genome ranged from 52.13% to 59.18%, with a unique mapping rate of 46.64% to 53.03% (Table 1). All the biological replicates showed strong correlations (Figure 4a). The R-value was 0.91, indicating that the differences between the groups were greater than those within the groups (Figure 4b). The majority of the unigenes (45.89–64.79%) had differential expression levels between 0 and 5 (Figure 4c).

### 2.5. Identification of Differentially Expressed Genes in Different Treatment Groups under Cd Treatment

**Figure 5:**
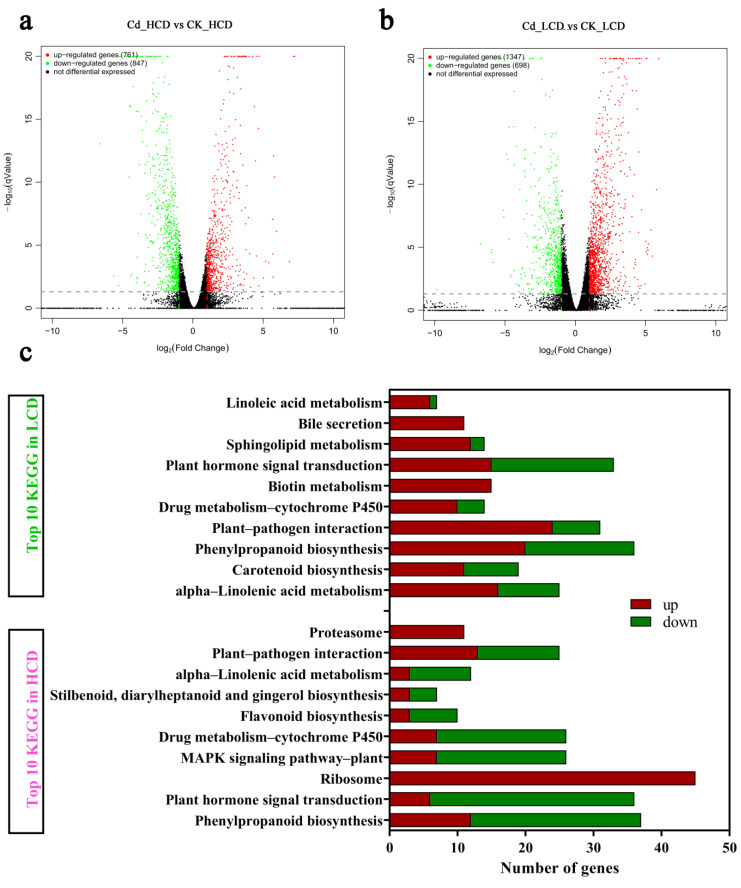
Differentially expressed genes in duckweed transcriptome under Cd treatment. (**a**) Expression difference volcano plot of HCD and (**b**) LCD. The horizontal axis is the expression difference fold-change (log_2_ (LCD/HCD)) value of genes between different sample groups. The vertical axis is the statistical significance *p*-value representing the change in gene expression. The smaller the *p*-value, the larger the −log (*p*-value), and the more significant the difference. Each point in the graph represents a gene: red indicates up-regulated genes, green indicates down-regulated genes, and black indicates non-differential genes. The dashed line represents the Q value for determining whether it is significant or not. The top part of the dashed line represents a significant difference (the closer to the top, the more significant it is) and the bottom half represents a non-significant difference. (**c**) Top 10 DEGs in KEGG enrichment for Cd_HCD vs. CK_HCD and Cd_LCD vs. CK_LCD.

To further investigate the molecular mechanisms of Cd^2+^ tolerance in the different strains, differentially expressed genes (DEGs) were identified throughout the course of Cd^2+^ treatment. As shown in Figure 5a, 1608 DEGs were found in HCD after Cd treatment compared to the control (Cd_HCD vs. CK_HCD), of which 761 DEGs were significantly up-regulated and 847 were significantly down-regulated. For the LCD strain, a total of 2045 DEGs were identified after Cd treatment versus the control (Cd_LCD vs. CK_LCD), of which 1347 DEGs were significantly up-regulated and 698 were significantly down-regulated (Figure 5b). Most obviously, there was a large difference in the number of up- vs. down-regulated genes (~2-fold difference) for HCD and LCD. HCD and LCD produced 632 DEGs after the Cd treatment (Figure 4d). A Kyoto Encyclopedia of Genes and Genomes (KEGG) enrichment analysis showed that 16 and 23 metabolic pathways in the HCD and LCD samples, respectively, responded significantly to Cd stress. Alpha-linolenic acid (ALA) metabolism, phenylpropanoid biosynthesis, plant hormone signal transduction, and drug-metabolism–P450 pathways were significantly annotated in both duckweed strains (Tables S1 and S2). For example, in plant hormone signal transduction, 36 DEGs (6 up-regulated and 30 down-regulated) were significantly annotated in Cd_HCD vs. CK_HCD, and 33 DEGs (15 up-regulated and 18 down-regulated) in Cd_LCD vs. CK_LCD (Figure 5c). Therefore, signal transduction may play an important role in the response to Cd stress in duckweed. These findings further indicate that the functional annotation of unigenes could be conducive to exploring the underlying mechanism of genotypic differences in Cd uptake by duckweed.

### 2.6. Functional Annotation of Unigenes and DEGs

**Figure 6:**
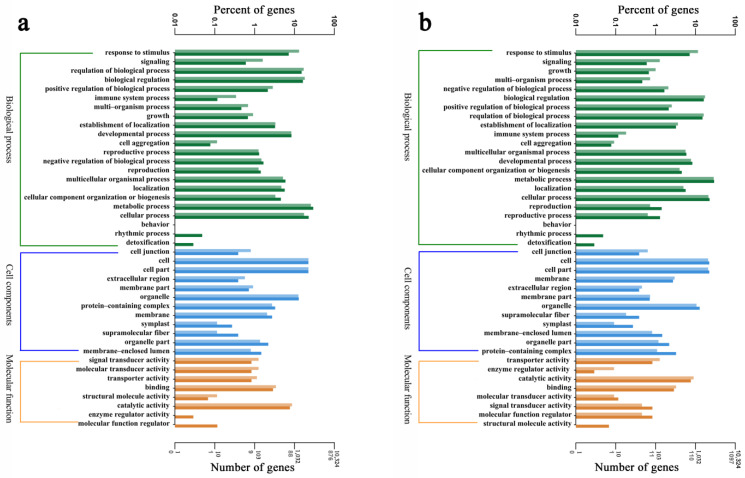
Gene ontology (GO) function classification for (**a**) HCD and (**b**) LCD. Lighter colours represent differential genes; darker colours represent all genes.

To elucidate the potential biological functions of the DEGs, a gene ontology (GO) enrichment analysis was performed using GO with *p* < 0.05. The GO enrichment analysis showed that 54 ontologies were significantly enriched in HCD, and 40 ontologies were significantly enriched in LCD (Figure S1). To understand the functions of these DEGs, GO annotations were used to classify the functions of predicted DEGs in duckweed under Cd stress, including the molecular function (MF), cellular components (CC), and biological process (BP) (Figure 6). In addition, GO terms, such as response to stimulus, vacuole, and monooxygenase activity, which may play key roles in abiotic stress, were found to be differentially ranked among the two strains (Figure 6, Figure S1).

### 2.7. Significantly Enriched Functions of Different Treatment Groups under Cd Treatment

**Figure 7:**
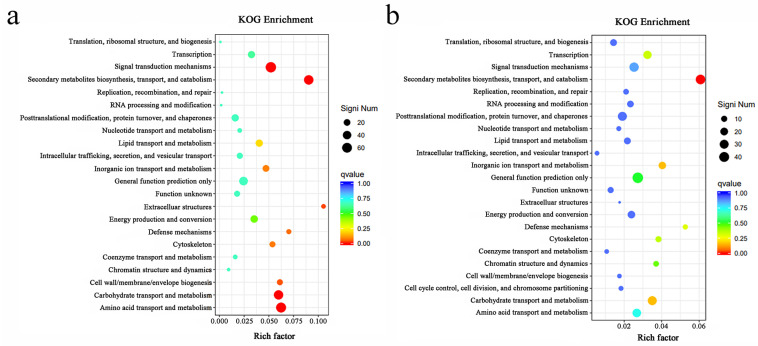
Scatter plot of significantly enriched functions for (**a**) HCD and (**b**) LCD. The vertical axis indicates the annotation information of the function. The horizontal axis indicates the Rich factor of the function. The size of Q-value is indicated by the dot colour: the smaller the Q-value is, the closer the colour is to red. The number of differential genes contained under each function is indicated by the dot size. Rich factor indicates the ratio of differential genes located in a functional entry to all genes located in that functional entry. Only the top 30 GOs with the highest level of enrichment were selected for plotting.

Functionally enriched genes were detected (Figure 7), and the Cd-tolerant HCD strain was mainly enriched in signal transport mechanisms, secondary metal biosynthesis transport and metabolism, carbohydrate transport and metabolism, and amino acid transport and metabolism. The Cd-sensitive LCD strain was mainly enriched in secondary metal biosynthesis, transport, and catabolism (Figure 7). These results indicate that signal transport mechanisms, secondary metal biosynthesis transport and metabolism, carbohydrate transport and metabolism, amino acid transport, and metabolism pathways play important roles in Cd tolerance in HCD.

### 2.8. Response of Starch Metabolism Pathway and Cell Wall Biosynthesis Pathway to Cd Stress in Duckweed

**Figure 8:**
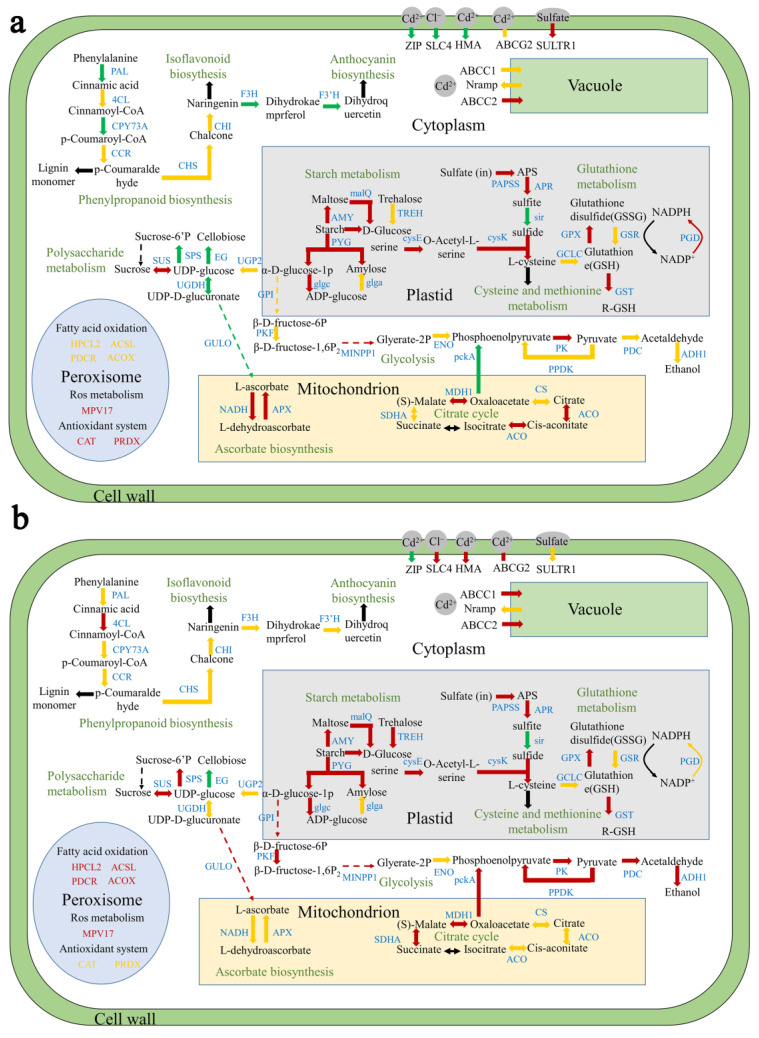
Cd detoxification mechanism in duckweed. Metabolic pathways of (**a**) HCD and (**b**) LCD. Red arrows represent up-regulation, green arrows represent down-regulation, and yellow arrows represent mixed regulation. Dotted lines indicate that genes whose expression did not change in this pathway or were not addressed in this study were omitted. Metabolic fluxes indicated by black arrows were not addressed in this study.

We analysed the profiles of genes involved in starch and sucrose metabolism, cell wall synthesis, and other glycan degradation pathways. Phenylalanine aminolyase (*PAL*), 4-coumarate-CoA ligase (*4CL*), and cinnamoyl coenzyme A reductase (*CCR*) were down-regulated under Cd stress. In addition, the enzyme expression of the cellulose degradation pathway was down-regulated under Cd treatment in both duckweed strains (Figure 8).

### 2.9. Sulphur and Glutathione Metabolism in response to Cd Stress

Cd affected the expression of sulphur- and glutathione-metabolism-related genes in the two studied duckweed strains. In both strains, 3′-phosphoadenosine 5′-phosphosulfate synthase and adenylyl-sulphate reductase (glutathione) (*APR*) were up-regulated in expression under Cd stress. Additionally, cysteine synthase (*cysK*) and glutathione peroxidase (*GPX*) were up-regulated under Cd stress (Figure 8).

### 2.10. Reactive Oxygen Species Metabolism Is Involved in High Cd Tolerance

The expression of MPV17 was up-regulated in both duckweed strains. In HCD, the genes encoding *CAT* and peroxidase (*PRDX*) showed an enhanced expression. The expression of genes encoding antioxidant enzymes did not change in the LCD strain. In addition, the genes encoding 2-hydroxyacyl-CoA lyase (*HACL1*), acyl-CoA oxidase *(ACOX*), 2,4-dienoyl-CoA reductase (*PDCR*), and long-chain acyl-CoA synthetase (*ACSL*) showed an enhanced expression in fatty acid oxidation (Figure 8). These results suggest that gene expression may differ under Cd stress in different duckweed strains.

### 2.11. Other Related Key Genes May Contribute to Cd Tolerance in Duckweed

Cd-tolerance-related genes were differentially expressed in response to Cd treatment in each strain (Figure 7 and Figure 8; Tables S3 and S4). Carbonic anhydrase (CA) and solute carriers SLC26, ABCB1, and ABCC2, and WRKY transcription factors, were significantly up-regulated in HCD under Cd stress. In LCD, SLC4, SLC26, ABCA3, ABCC1, and ABCG2 were up-regulated. In addition, WRKY was down-regulated in LCD. In addition, many chaperones were annotated in both LCD and HCD (Tables S3 and S4), suggesting these chaperones are important in responses to Cd stress. 

### 2.12. DNA Repair in Response to Cd Stress

**Figure 9:**
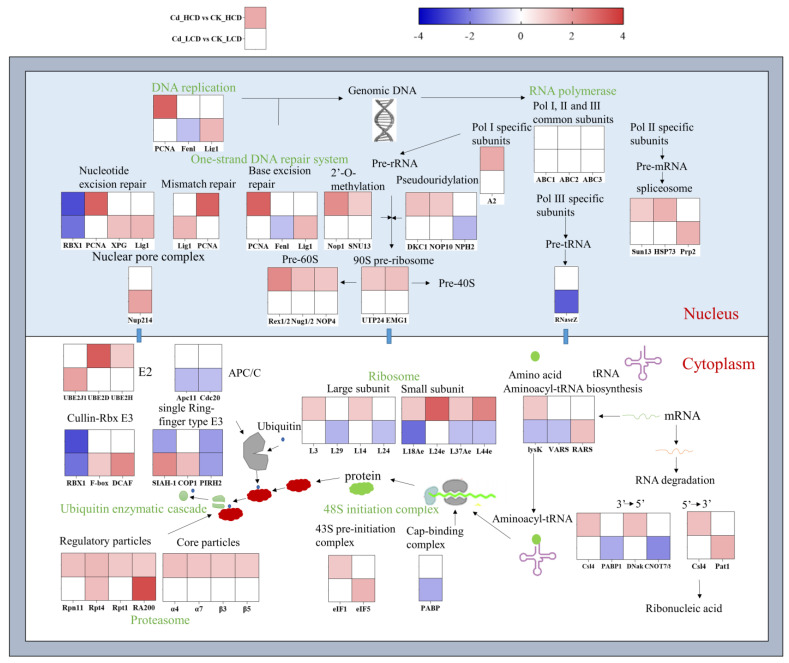
Response of basal biological processes under Cd stress in duckweed. Several biological processes as well as related protein complexes in response to Cd are presented in the diagram. In the diagram, the upper blocks are the nucleus and the lower blocks are the cytoplasm. Black arrows indicate process directions. Cd_HCD vs. CK_HCD and Cd_LCD vs. CK_LCD represent the gene expression of HCD and LCD at 12 h of the 0.5 mg/L Cd^2+^ treatment, respectively.

To explore the possible molecular mechanisms mediating Cd tolerance in duckweed, we investigated the expression of the genes involved in replication, transcription, and translation (Figure 9). Proliferating cell nuclear antigen (*PCNA*) expression was up-regulated in HCD but was unchanged in LCD. Flap endonuclease-1 (*Fen1*) was down-regulated in LCD. Additionally, the expression of DNA ligase 1, which is involved in DNA replication, was up-regulated in LCD.

### 2.13. RNA and Protein-Related Biological Processes in Response to Cd Stress

As shown in Figure 9, only A2 expression in the Pol I subunit of HCD was up-regulated under Cd stress. The expression of other genes in LCD remained unchanged. In LCD, the expression of the H/ACA ribonucleoprotein complex subunit 2 (*NPH2*) transcript associated with ribosome processing was down-regulated. In HCD, the expression of the exosome complex components CSL4 (*Csl4*) and Rrp43 was up-regulated. In LCD, polyadenylate-binding protein (*PABP1*), which prevents mRNA degradation, was down-regulated in LCD. In HCD, the 48S initiation complex and ribosome biosynthesis were up-regulated, whereas the 48S initiation complex and ribosome biosynthesis were down-regulated or almost unchanged in LCD (Figure 9).

### 2.14. Alpha-Linolenic Acid Metabolism in Response to Cd Stress

**Figure 10:**
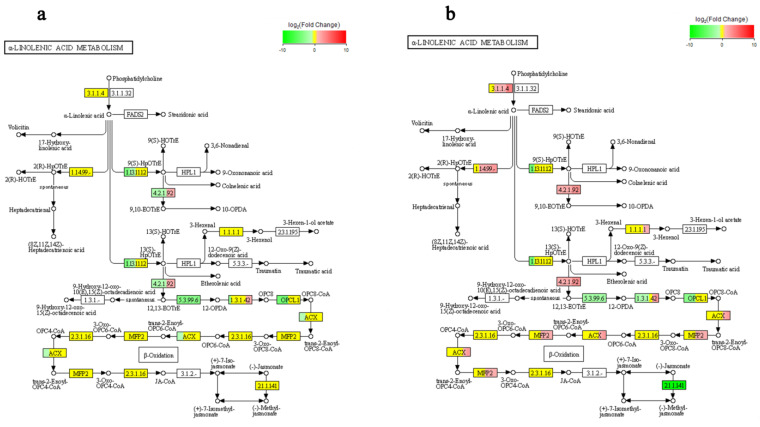
Alpha-linolenic acid metabolism pathway in (**a**) HCD and (**b**) LCD. Red represents upregulation, green represents down-regulation, and yellow represents mixed regulation. The size of the colour block represents the proportion of the corresponding gene in the gene product.

We observed the differential expression of genes related to the ALA metabolic pathway in the two duckweed strains (Figure 10).

## 3. Discussion

Heavy metal pollution is a major environmental stress factor that affects plant growth and development [50]. The heavy metal Cd inhibits various physiological processes in plants including growth, photosynthesis, chlorophyll content, and antioxidation [22]. There is a great intra-species variation in Cd tolerance in duckweed. Similar results have been reported by Chen et al. [20]. The chlorophyll content gradually decreased with increasing Cd treatment time (Figure 1d,e,g,h). This is in agreement with the findings of Szopiński et al. [51]. The chlorophyll content in HCD was higher than that in LCD at different Cd stress times (Figure 1f). In *Thlaspi fendleri*, Cd-sensitive strains have been reported to decrease the chlorophyll content to a greater extent than Cd-tolerant strains [52]. This may be because chlorophyll synthesis was less inhibited in the HCD strain than in the LCD strain [51,52]. The threshold value for Cd-hyper-enriched plants is typically 100 mg/kg (DW) [53]. The concentration of Cd in HCD increased first and then decreased with time. It decreased probably due to new plant growth at the lower Cd levels that results after Cd levels rapidly decrease from the uptake into HCD plants. Both HCD and LCD were Cd-hyper-enriched plants. The results showed that the removal efficiency of HCD was better than that of LCD and demonstrated that the effect of Cd concentration could be increased by extending the time when used for environmental remediation.

A negative effect of Cd accumulation in plants is the production of excess reactive oxygen species (ROS) [54]. CAT, POD, and SOD scavenge and reduce oxidative damage caused by excess ROS, and are key enzymes used by various plants to adapt to the environment. Hence, they are key indicators of the antioxidant capacity of plants [55,56]. In a previous study on Cd-stressed wheat, antioxidant enzyme activity increased with increasing Cd stress duration, which is consistent with the results of the present study [57]. Glutathione S-transferase (GST) is the main system for exogenous detoxification and cellular resistance to damage in plants [47,48,49]. The GST activity of the HCD strain was higher than that of the LCD strain at different time points (Figure 3k). This is probably because HCD absorbed more cadmium than LCD early in the treatment; the concentration of Cd in the medium was reduced and HCD was subjected to lower Cd stress. These results indicate that GST may be the key factor contributing to antioxidation in HCD and that the HCD strain was able to produce more GST to alleviate Cd toxicity and cellular damage. Additionally, the antioxidant enzyme system of duckweed plays an important role in coping with Cd-induced oxidative stress.

Starch is a resource held in plants that remobilizes its starch reserves in response to abiotic stresses such as a drought, a high salinity, and harsh temperatures, releasing energy and sugars and thereby aiding in stress reduction [58]. Glycolysis and the tricarboxylic acid (TCA) cycle are the major pathways of starch metabolism and tend to be overexpressed, in part, under Cd stress, owing to a high energy demand (Figure 8). Excess alcohol synthesised during anaerobic respiration has toxic effects on plants [59]. The expression of biosynthesis ethanol genes was up-regulated in Cd-sensitive LCD, which might be partly responsible for the severe toxicity exhibited by this strain (Figure 8). Phenolic compounds (e.g., phenolic acids and flavonoids) are an important class of plant secondary metabolites that can scavenge ROS [60]. The phenylpropanoid biosynthetic pathway is activated under abiotic stress conditions (e.g., drought, heavy metals, and salinity), leading to the accumulation of various phenolic compounds [60]. In the present study, the expression of enzymes related to this pathway, such as PAL, 4CL, and CCR, was down-regulated under Cd stress. Similar results were reported in *L. punctata* 6001 [42]. The cell wall is thought to be a site of Cd binding and is involved in Cd accumulation in *Sedum alfredii* and rice [61,62]. In contrast, enzyme expression in the cellulose degradation pathway was down-regulated under Cd treatment in the two duckweed strains, suggesting that the duckweed cell wall may have been involved in Cd binding. Flavonoids protect plants against various biotic and abiotic stresses [63]. Flavonoid-related biosynthetic genes in HCD and LCD were down-regulated or remained constant, implying that duckweed flavonoid compounds did not function under Cd stress (Figure 8).

It has been shown that the expression of genes related to sulphur metabolism and glutathione metabolism is associated with Cd tolerance in plants [64]. 3′-phosphoadenosine 5′-phosphosulfate synthase and *APR* are involved in sulphate activation and its reduction to sulphide, and the two enzyme genes are up-regulated under Cd stress (Figure 8). Similar results have been reported for *Medicago sativa* [64]. Sulphide is synthesised as cysteine with O-acetyl serine by the action of *cysK* and the expression of this enzyme is up-regulated according to transcriptomic data. Glutathione is a reducing agent that helps remove ROS and is produced from glutathione disulphide (GSSG) by *GPX* and glutathione reductase (GSR) [65]. Glutathione peroxidase expression was up-regulated under Cd stress, implying that GSH and GSSG cycling may be enhanced to improve ROS scavenging in duckweed (Figure 8). The results showed that the two duckweed strains under Cd stress had similar expression levels of genes related to sulphur and glutathione metabolism. Glutathione is converted to R-S-GSH by glutathione sulfotransferase for Cd^2+^ chelation in duckweed, which further improves its tolerance to Cd (Figure 8). 

Peroxisomes are metabolic organelles that are mainly involved in lipid metabolism, ether lipid synthesis, and ROS metabolism [66]. MPV17 is a protein that has been suggested to be involved in the metabolism of reactive oxygen species [67]. Wi et al. [68] found that the overexpression of the *MPV17* gene in *Arabidopsis* increased resistance to stress. The expression of MPV17 was up-regulated in both duckweed strains, suggesting that MPV17 may be involved in Cd tolerance in duckweed. In HCD, the genes encoding *CAT* and *PRDX* also showed an enhanced expression, indicating that antioxidant enzymes may contribute to cell survival under Cd^2+^ stress. The expression of the genes encoding antioxidant enzymes did not change in the LCD strain. In addition, in LCD, the genes encoding *HACL1*, *ACOX*, *PDCR*, and *ACSL* showed an enhanced expression during fatty acid oxidation, whereas their expression levels were not up-regulated in HCD (Figure 8). The results showed that different strains of duckweed responded differently to Cd stress, which may explain why HCD plants were damaged to a lesser extent than LCD plants.

Carbonic anhydrase is a ubiquitous metalloenzyme involved in respiration, calcification, and biosynthesis that readily binds metal ions at the active site [69]. Caricato et al. [70] found that CA activity and protein expression were enhanced in *Mytilus galloprovincialis* under Cd stress. This is in agreement with the results of the present study, where CA gene expression was significantly up-regulated in strain HCD under Cd stress and was much higher than that in strain LCD (Tables S3 and S4). Studies have shown that the solute carriers *SLC4* and *SLC26* superfamilies play an important role in the process of oxidative stress [71]. In this study, SLC26 expression in HCD cells was up-regulated in response to Cd stress. In LCD, both *SLC4* and *SLC26* were up-regulated. It has been shown that the ATP-binding cassette transporter (ABC transporter) was associated with Cd tolerance in a plant [72]. In the present study, the expression of ABC transporter protein superfamily genes was up-regulated under Cd stress in both duckweed strains. Among these, the expression of *ABCB1* and *ABCC2* was up-regulated in HCD. Similar results have been reported for *Ophiopogon japonicas* [73]. In addition, the expression of *ABCA3*, *ABCC1*, and *ABCG2* was up-regulated in LCD (Figure 8; Tables S3 and S4). Heat shock proteins (HSPs) are a class of stress proteins synthesised by organisms exposed to abiotic stresses that have been shown to protect cells from oxidative damage and apoptosis triggered by Cd exposure [74]. HSPs were found to play a significant role in protecting plants from adverse environments [75]. *HSP90* and *HSP70* overexpression contribute to the prevention of Cd toxicity [76]. In total, 24 and 13 genes were up-regulated in HCD and LCD groups, respectively. The transcription factor (TF) WRKY is known to respond to different biotic and abiotic stresses [77]. In addition, *WRKY* overexpression was found to significantly promote the uptake and accumulation of Cd in poplars [78]. In our study, *WRKY22* and *WRKY29* were up-regulated in the HCD group and down-regulated in LCD, which indicated that WRKY TFs may play a unique role in Cd tolerance. Therefore, these may be important factors for the better adaptation of the HCD strain to Cd stress.

Cadmium is known to affect plant cell proliferation and differentiation, cell cycle progression, DNA repair, DNA synthesis, apoptosis, and other BPs in a variety of ways [18]. Therefore, Cd stress is one of the factors contributing to genomic instability in duckweed [79]. DNA repair mainly includes mismatch repair (MMR), nucleotide excision repair (NER), and base excision repair (BER) [80]. In the present study, the relevant unigenes involved in the three repair systems were significantly altered in response to Cd stress. The upregulation of PCNA, a key gene in DNA repair and cell cycle regulation, contributes to the deleterious effects of Cd exposure [81]. Proliferating cell nuclear antigen expression was up-regulated in the HCD group but remained unchanged in the LCD group (Figure 9). The expression of flap endonuclease-1 (Fen1), a flap endonuclease involved in cutting the base of single-stranded flaps, was down-regulated in LCD [82]. In addition, the expression of DNA ligase 1, which is involved in DNA replication, was up-regulated in the LCD strain, suggesting a possible mechanism for coping with the oxidative damage under Cd stress (Figure 9). These results indicate that DNA replication and repair systems appear to have similar responses to Cd^2+^, suggesting that the maintenance of genomic stability plays a central role in resistance to a high mutagenicity triggered by Cd^2+^ in plants. This may be because the two duckweed strains respond differently to Cd^2+^ toxicity.

RNA- and protein-related BPs are seriously threatened due to the cytotoxicity of Cd^2+^ [83]. Gene expression in the rRNA, mRNA, and tRNA synthesis pathways associated with protein synthesis was altered under Cd stress (Figure 9). The rRNA, mRNA, and tRNA precursors were synthesised using RNA polymerases (Pol) I, II, and III. As shown in Figure 9, only A2 expression in the Pol I subunit of the HCD strain was up-regulated under Cd stress. The expression of other genes in the LCD strain remained unchanged. Based on the response of RNA Pol I, the maturation and translocation of rRNA in HCD appeared to be induced by Cd^2+^, ultimately favouring ribosome biosynthesis. Similar results were reported by Lv et al. [83] for *Landoltia punctate*. The expression of *NPH2* transcripts related to ribosome processing was down-regulated in LCD (Figure 9). According to the RNA Pol II and III results, the genes synthesised by mRNA and tRNA remained essentially unchanged. The results showed that rRNA biosynthesis was enhanced in HCD, which facilitated the further expression of the duckweed genome. Considering that significant Cd-induced oxidative damage was found in LCD, we suggest that rRNA biosynthesis-related genes may be one of the factors of Cd tolerance in duckweed.

However, there may be a complex mechanism underlying the Cd^2+^ stress response during mRNA splicing and degradation. In eukaryotes, introns in pre-mRNAs are removed by spliceosomes. In this study, the transcript levels of DEGs involving spliceosomes were up-regulated in both duckweed strains after Cd^2+^ treatment (Figure 9). In addition, mRNA degradation in eukaryotes occurs via two mechanisms: First, the hydrolytic shortening of poly(A) occurs, followed by degradation by 5′-end decapitation and 5′→3′ directional nucleic acid exonuclease action. Second, the hydrolysis of poly(A) occurs, followed by the 3′→5′ degradation of the polysome exosome complex. HCD and LCD showed different response patterns for RNA degradation. In the HCD group, *Cls4* and *Rrp43* gene expression were up-regulated. In LCD, *PABP1*, which prevents mRNA degradation, was down-regulated. The results show that RNA degradation may be induced by the Cd treatment in both duckweed strains. The 48S initiation complex, ribosomes, and aminoacyl-tRNA, including modified tRNA, are the basic components of cellular protein factories. Protein production and ubiquitin-mediated protein hydrolysis changes exist in response to Cd stress [83]. In HCD, the 48S initiation complex and ribosome biosynthesis were up-regulated, whereas the 48S initiation complex and ribosome biosynthesis were down-regulated or almost unchanged in LCD. The ubiquitin 26S proteasome system plays an important role in hormone signalling, transcriptional regulation, and plant response to environmental challenges [84]. In HCD, the expression of APC/C, Cullin-Rbx E3, and single ring-finger type E3 was down-regulated in response to Cd stress, except for the expression of the ubiquitin-binding enzyme E2, which was up-regulated. LCD showed different response mechanisms, indicating that Cd stress has its own specificity for the response mechanisms of Cd-tolerant and Cd-sensitive strains.

The omega-3 polyunsaturated fatty acid ALA is extracted from plant sources and has been shown to be one of the anti-inflammatory and antioxidant agents [85]. Oral ALA was demonstrated to prevent Cd-induced oxidative stress, neuroinflammation, and neurodegeneration in the mouse brain [85]. In this study, the expression of the ALA metabolic pathway was largely down-regulated in Cd-tolerant HCD, suggesting that HCD may have more ALA and thus be better equipped to cope with Cd-induced oxidative stress compared to LCD. Conversely, the ALA metabolic pathway of Cd-sensitive LCD was largely up-regulated, and ALA may have been metabolised more through the metabolic pathway. These results suggest that α-linolenic acid metabolism may be involved in the high Cd tolerance of duckweed. Another alternative explanation for LCD is that greater oxidative damage caused a greater need to metabolize damaged lipids.

Although we found that a lot of DEGs may be related to duckweed Cd tolerance, due to different read numbers, different rRNA expression, and uncertainty in RNA preparation, the ratio used to calculate DE can be distorted, and the TPM cannot be normalized to some extent [86]. Therefore, it is necessary to verify the function of candidate genes through gene overexpression and gene knockout in future studies. The results of this study lay a foundation for the study of plant Cd tolerance.

## 4. Materials and Methods

### 4.1. Isolation and Culture of Duckweed

In this study, two duckweed strains with a Cd-hyper-enrichment capacity were used as experimental materials. Two duckweed strains, *Lemna minor* 0009 (HCD, a high-Cd-tolerance cultivar) and *Lemna minor* 0010 (LCD, a low-Cd-tolerance cultivar), were obtained from the Duckweed Germplasm Bank of the College of Life Sciences at Guizhou University. Before experimental treatment, a Hoagland’s culture solution (containing 1.5% sucrose) was used to pre-culture the duckweed strains [87]. The duckweed plants were cultured at 25 °C under a 16/8 h light–dark cycle and light intensity of 5000 lx for 7 d. After the pre-culture, duckweed plants with good growth characteristics were selected for subsequent experimental treatments.

### 4.2. Experimental Design

**Figure 11:**
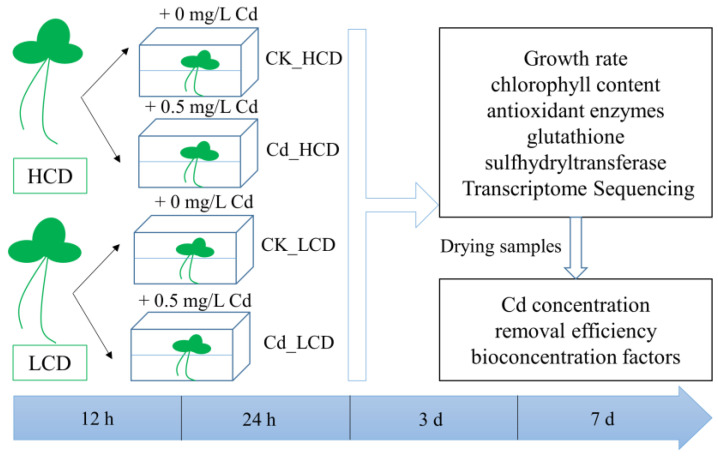
Experimental design.

Weigh 1.2 g (fresh weight) of HCD and LCD in a 13 × 8.2 × 5.3 cm box, respectively. To investigate the tolerance mechanism of Cd in duckweed, 0.5 mg·L^−1^ of Cd^2+^ was prepared with a CdCl_2_ solution (aladdin, Shanghai, China) and treated with duckweed. Among them, HCD and LCD were treated with 0.5 mg/L of Cd^2+^ for different times at 25 °C under a 16/8 h light–dark cycle, 75% humidity, and light intensity of 5000 lx. Conduct three biological replicates per treatment. The groups were distinguished as follows: HCD control group (CK_HCD, no Cd^2+^ added); HCD treatment group (Cd_HCD, 0.5 mg/L of Cd^2+^ added); LCD control group (CK_LCD, no Cd^2+^ added), and LCD treatment group (Cd_LCD, 0.5 mg/L of Cd^2+^ added) (Figure 11). Duckweed material was collected at 0 h, 12 h, 3 d, and 7 d to determine plant growth, chlorophyll content, Cd concentration, and antioxidant enzyme activity. The collected samples were immediately frozen in liquid nitrogen and kept at −80 °C for RNA-seq.

### 4.3. Determination of Growth Rate

Duckweed samples were collected at 12 h, 24 h, 3 d, and 7 d. After the treated duckweed samples were harvested, they were rinsed with ultrapure water and blotted using filter paper sheets. The fresh weights of duckweed samples were measured and recorded. Three biological replicates were used for each treatment, and each sample was cultivated in a separate box. The growth rate (GR) of duckweed was calculated according to Formula (1) [44]:

(1) $$\mathrm{GR}=\frac{\Delta W}{T}=\frac{W_{T}-W_{0}}{T}$$

where GR is the growth rate in g/d, W is the change in the fresh weight of duckweed after different treatment times (g), T is the treatment period (d), W_0_ is the fresh weight on the first day (g), and W_7_ is the fresh weight on day 7 (g).

### 4.4. Determination of Chlorophyll Content

After aspirating the water on the surface of the duckweed, 0.1 g of the sample was weighed in a 10 mL centrifuge tube, placed in a −20 °C freezer for 1 h, removed to add 2 mL of 95% ethanol, preheated to 50 °C, shaken thoroughly, and placed in the dark at room temperature for 3 h. The supernatants were then collected. Absorbances of the chlorophyll pigments in the collected supernatants were measured at 663 and 645 nm using a full-wavelength Multiskan microplate photometer (Thermo Scientific Multiskan FC, Shanghai, China), and the chlorophyll content was calculated according to Equations (2)–(4).

Chl a = 12.7A_663_ − 2.69A_645_ (2)

Chl b = 22.9A_645_ − 4.68A_663_ (3)

Chl = Chl a + Chl b
(4)

where Chl a is the concentration of chlorophyll a (mg/L), Chl b is the concentration of chlorophyll b (mg/L), Chl is the concentration of chlorophyll (mg/L), A_663_ is the absorbance of the chlorophyll solution at 663 nm, and A_645_ is the absorbance of the chlorophyll solution at 645 nm converted to chlorophyll content per gram of fresh leaves (mg/g) based on the chlorophyll concentration in the extracts.

### 4.5. Determination of Cd Content in Duckweed Samples and Culture Media

The duckweed was rinsed sequentially with flowing tap water and ultrapure water, and the fresh weight was recorded after filter paper was blotted dry. The fresh duckweed samples were then placed in an oven at 60 °C to dry overnight until their weight was constant. Samples were ground into a powder and placed in test tubes for measurement. First, 0.1 g of sample powder was weighed in the digestion tube and 2 mL of concentrated nitric acid was added overnight. Then, 4 mL of concentrated nitric acid was added and mixed thoroughly. Digestion progressed at 280 °C for 4 h (GB/T 23739–2009). A blank control was used for each digestion stage to eliminate possible errors. After digestion, the remaining digestion solution was cooled to room temperature in a digestion tube and washed with deionised water. Then, the volume was fixed at 50 mL for the analysis. For each treatment, 50 mL of the liquid medium was obtained and centrifuged at 3500 rpm for 10 min, and the supernatant was placed in a refrigerator at 4 °C for measurement. A flame atomic spectrophotometer (Analytik Jena AG NovaAA 400P, Jena, Germany) was used to detect Cd [88,89].

Cd concentration in duckweed was calculated according to Formula (5) [90], the removal efficiency of Cd from water bodies by duckweed was calculated according to Formula (6), and bioconcentration factors (BCFs) were the concentration ratios of heavy metals in plant tissues to the aqueous environment and are commonly used to evaluate the accumulation trends of heavy metals in organisms [91]. BCF was calculated according to Formula (7).

(5) $$M=\left(C_{t}-C_{0}\right)V/m\;$$

where M is the Cd content in the sample per unit weight (mg/kg), C_t_ is the Cd content in the sample digestion solution (mg/L), C_0_ is the Cd content in the blank digestion solution (mg/L), V is the total volume of the sample digestion solution (mL), and m is the total amount of dry powder weighed during digestion (g).

(6) $$R=\left(\frac{C_{i}-C_{f}}{C_{i}}\right)\times 100\%$$

where R is the removal efficiency of Cd from water by duckweed, C_i_ is the initial Cd concentration (mg/L), and C_f_ is the residual Cd concentration after treatment (mg/L).

(7) $$R_{\mathrm{BCF}}=C_{p}/C_{h}$$

where R_BCF_ is the bioconcentration factor, C_p_ is the concentration of Cd in the plant tissues (mg/kg), and C_h_ is the final concentration of Cd in the culture solution (mg/L).

### 4.6. Determination of Antioxidant Enzyme Activity and Glutathione Sulfotransferase Activity

To prevent enzyme inactivation, we weighed 0.1 g of duckweed tissue that had been frozen in liquid nitrogen. Then, 1 mL of the extraction solution was added, and the material was homogenised in an ice bath. The supernatant was removed after centrifugation at 8000 rpm for 10 min at 4 °C and put on ice for measurement [92,93]. Superoxide dismutase (SOD, EC 1.15.1.1), peroxidase (POD, EC 1.11.1.7), catalase (CAT, EC 1.11.1.6), and glutathione thioltransferase (GST, EC 2.5.1.18) activities were measured in 1 mL of the supernatant using specific kits according to the manufacturer’s instructions (Solarbio, Beijing, China).

### 4.7. RNA Extraction and cDNA Library Construction

For the transcriptome analysis, HCD or LCD was treated with 0 and 0.5 mg/L of Cd^2+^ for 12 h, and duckweed samples were collected for total RNA extraction. Plant tissues were frozen in liquid nitrogen. Total RNA was extracted using a Total RNA Extractor (Trizol) kit (Sangon Biotech) (Total RNA Extractor, Trizol), and RNA concentration was measured using a Qubit2.0 RNA assay kit (Life). RNA integrity and genomic contamination were detected with agarose gel (PCR instrument, T100™ Thermal Cycler). Using the 3′ polyA structure of messenger RNA and related molecular biology techniques, we performed experiments on the complete total RNA of 12 *Lemna minor* samples, including mRNA isolation, fragmentation (Hieff NGS™ MaxUp Dual-mode mRNA Library Prep Kit for Illumina^®^, YEASEN), double-stranded cDNA synthesis (low-temperature centrifuge, Thermo Scientific Sorvall Legend Micro 21R), cDNA fragmentation modification, magnetic bead purification and fragmentation sorting (Hieff NGS^®^ DNA Selection Beads, YEASEN), library amplification, and other processes. The recovered DNA was accurately quantified using a Qubit DNA Assay Kit to facilitate mixing in equal amounts at a 1:1 ratio for sequencing afterwards. After testing and quality control, we finally obtained sequencing libraries that were suitable for “paired-end” 2 × 150. Raw image data files from “paired-end” 2 × 150 were converted into raw sequenced reads with a CASAVA Base Calling analysis. A quality assessment of the raw sequenced data was performed using FastQC software. Relatively accurate clean reads were obtained with quality control using Trimmomatic software. The *Lemna minor* genome was used as the reference sequence (genome ID: 27408). After QC, the sequenced sequences were compared with the reference genome using HISAT2, and the comparison results were counted using RSeQC. We treated the samples with different Cd concentrations, collected the samples and sent them to Sangon Biotech (Shanghai) Co., Ltd. (Shanghai, China), and performed library construction and RNA-seq for each sample separately on an Illumina Hiseq™ platform.

### 4.8. RNA-seq Data Processing

Raw reads of the transcriptome dataset were processed by Sangon Biotech (Shanghai) Co. The reference genome was used as the reference sequence, and the sequences after quality control were mapped with the reference genome using HISAT2. The comparison results were counted using RSeQC. Gene function annotations were selected from the following authoritative databases: Gene Ontology (GO), Kyoto Encyclopedia of Genes and Genomes (KEGG), homologous-protein-clustering NCBI COG/KOG, UniProt, Ensembl, Biomart, protein-family PFAM, CDD, STRING, NCBI NT, and NCBI NR.

### 4.9. Statistical Analysis

Statistical analyses were performed using IBM SPSS Statistics 26 and GraphPad Prism software. *p*-Values were calculated with a one-way analysis of variance (ANOVA), and the multiple t-test method was used for analytical comparison to determine the significance of differences between treatment means (*p* < 0.05). Data are means ± SE, replicated three times.

## 5. Conclusions

For time reasons, we did not perform a functional validation of the screened-in genes of interest. Our physiological and transcriptome investigations of two duckweed strains demonstrated significant differential effects of Cd stress on growth rate and chlorophyll content. A significant intra-species variation was observed in Cd tolerance and Cd removal efficiency. Specifically, HCD showed a significantly higher removal efficiency than LCD. The antioxidant enzyme systems of the two duckweed strains were also affected differently under Cd stress, and greater Cd-induced oxidative damage was observed in LCD. GST activity was higher in the HCD strain than in the LCD strain at all time points. In addition, based on the transcriptomic analysis, there were 1608 and 2045 DEGs in the HCD and LCD duckweed strains, respectively, under Cd stress, with significant genotypic differences. Starch metabolism, sulphur metabolism, and ROS pathways were activated, and the expression of glutathione-synthesis-related genes was up-regulated to eliminate the effects of Cd-induced ROS. The gene expression of the genetic central dogma is triggered within 12 h in response to the Cd exposure. Genes involved in Cd uptake and transport, such as ABC transporter proteins, were actively expressed under Cd stress. These results improve our understanding of the potential mechanisms underlying Cd tolerance in duckweed at the transcriptomic level. They also lay the foundation for our future research on Cd-tolerance-related pathways and genes.

## Figures and Tables

**Table 1:** Quality and read mapping statistics for transcriptome data from HCD and LCD genotypes of duckweed.

Group	CK_HCD	Cd_HCD	CK_LCD	Cd_LCD
Raw Reads Count	43,835,874	43,830,797	46,377,103	57,237,400
Clean Reads Count	41,233,077	41,778,800	43,915,528	54,521,819
Q20 Bases Ratio (%)	97.28%	97.58%	97.26%	98.06%
Q30 Bases Ratio (%)	89.87%	90.36%	89.59%	92.60%
GC Bases Ratio (%)	55.00%	54.71%	54.18%	54.41%
Total mapped	20,812,521 (52.13%)	22,147,187 (54.56%)	24,396,197 (56.99%)	31,782,882 (59.18%)
Uniquely mapped	18,634,487 (46.64%)	19,784,897 (48.76%)	21,864,070 (51.08%)	28,483,426 (53.03%)

## Data Availability

Data available on request due to restrictions eg privacy or ethical. The data presented in this study are available on request from the corresponding author.

## Supplementary Materials

The following supporting information can be downloaded at: https://www.mdpi.com/article/10.3390/ijms241512157/s1.
