# Supplementary material for: Transcriptomic and Functional Analyses of Two Cadmium Hyper-Enriched Duckweed Strains Reveal Putative Cadmium Tolerance Mechanisms
Source: Int J Mol Sci. 2023 Jul 29;24(15):12157. doi: 10.3390/ijms241512157 (PMC10418380; doi:10.3390/ijms241512157)
Supplement: Supplementary file 1 [file ijms-24-12157-s001.zip › ijms-2363198-supplementary.pdf]

International Journal of *Molecular Sciences*

MDPI

Article

Transcriptomic and Functional Analyses of Two Cadmium Hyper-enriched Duckweed Strains Reveal Putative Cadmium Tolerance Mechanisms

Gui-Li Yang [1,2], Lei Huang [3], Xiao Yang [1], Zhu Li [1], Hai-Min Liao [1], Kang Mao [2], Zhao-Ju Liu [1], He-Yan Geng [1], Qin Cao [1] and Ai-Juan Tan [1,*]

1 Key Laboratory of Plant Resource Conservation and Germplasm Innovation in Mountainous Region (Ministry of Education), Collaborative Innovation Center for Mountain Ecology & Agro-Bioengineering (CICMEAB), College of Life Sciences/Institute of Agro-Bioengineering, Guizhou University, Guiyang 550025, China; glyang3@gzu.edu.cn (G.-L.Y.); yangxiao98103@163.com (X.Y.); zhuliluck@163.com (Z.L.); lhaimin@163.com (H.-M.L); lzhju6@163.com (Z.-J.L); hygeng2022@163.com (H.-Y.G); caoqin9909@163.com (Q.C.); ajtan@gzu.edu.cn (A.-J.T)
2 Institute of Geochemistry, Chinese Academy of Sciences, Guiyang 550081, China; maokang@mail.gyig.ac.cn (K.M.)
3 The Key Laboratory of Chemistry for Natural Products of Guizhou Province and Chinese Academy of Sciences, Guiyang 550014, China; xinyanghuanglei@163.com (L.H.)
* Correspondence: ajtan@gzu.edu.cn; Tel.: +86-1376-513-6919

Citation: Yang, G.-L.; Huang, L.; Yang, X.; Li, Z.; Liao, H.-M.; Mao, K.; Liu, Z.-J.; Geng, H.-Y.; Cao, Q.; Tan, A.-J.; et al. Transcriptomic and Functional Analyses of Two Cadmium Hyper-enriched Duckweed Strains Reveal Putative Cadmium Tolerance Mechanisms. *Int. J. Mol. Sci.* **2023**, *24*, 12157. https://doi.org/10.3390/ijms241512157

Academic Editor: Jozef Kovacik

Received: 8 April 2023
Revised: 26 July 2023
Accepted: 27 July 2023
Published: 29 July 2023

Copyright: © 2023 by the authors. Licensee MDPI, Basel, Switzerland. This article is an open access article distributed under the terms and conditions of the Creative Commons Attribution (CC BY) license (https://creativecommons.org/licenses/by/4.0/).

Supplementary Materials:

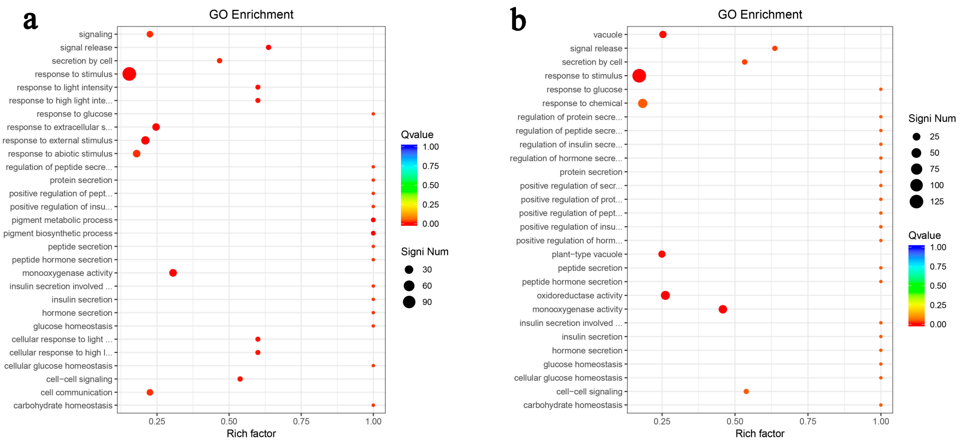

Figure S1. Scatter plot of GO enrichment with significant differences in expression for (a) HCD and (b) LCD.

Table S1. KEGG enrichment analysis of HCD

ID	Description	Significant	Annotated	P-value	Q-value
ko00940	Phenylpropanoid biosynthesis	37/481	165/6968	7.55E-11	1.33E-08
ko04075	Plant hormone signal transduction	36/481	180/6968	3.91E-09	3.43E-07
ko03010	Ribosome	45/481	300/6968	4.40E-07	2.58E-05
ko04016	MAPK signaling pathway - plant	26/481	139/6968	2.30E-06	0.000101
ko00982	Drug metabolism - cytochrome P450	13/481	43/6968	3.69E-06	0.00013
ko00941	Flavonoid biosynthesis	10/481	30/6968	1.91E-05	0.000559
ko00945	Stilbenoid, diarylheptanoid and gingerol biosynthesis	8/481	20/6968	2.92E-05	0.000733
ko00592	alpha-Linolenic acid metabolism	12/481	45/6968	3.59E-05	0.000789
ko04626	Plant-pathogen interaction	25/481	157/6968	6.41E-05	0.001252
ko03050	Proteasome	11/481	45/6968	0.000178	0.003136
ko04727	GABAergic synapse	9/481	33/6968	0.000285	0.004213
ko00980	Metabolism of xenobiotics by cytochrome P450	10/481	40/6968	0.000288	0.004213
ko04141	Protein processing in endoplasmic reticulum	26/481	184/6968	0.000341	0.004606
ko03008	Ribosome biogenesis in eukaryotes	14/481	82/6968	0.001303	0.016355
ko00780	Biotin metabolism	10/481	49/6968	0.001591	0.018641
ko00908	Zeatin biosynthesis	5/481	16/6968	0.003542	0.038914
ko00910	Nitrogen metabolism	13/481	84/6968	0.004665	0.048242

Table S2. KEGG enrichment analysis of LCD

ID	Description	Significant	Annotated	P-value	Q-value
ko00592	alpha-Linolenic acid metabolism	25/583	45/6968	4.55E-16	9.35E-14
ko00906	Carotenoid biosynthesis	19/583	48/6968	2.91E-09	2.98E-07
ko00940	Phenylpropanoid biosynthesis	36/583	165/6968	5.55E-08	3.80E-06
ko04626	Plant-pathogen interaction	31/583	157/6968	4.52E-06	0.000212
ko00982	Drug metabolism - cytochrome P450	14/583	43/6968	5.63E-06	0.000212
ko00780	Biotin metabolism	15/583	49/6968	6.19E-06	0.000212
ko04075	Plant hormone signal transduction	33/583	180/6968	1.18E-05	0.000347
ko00600	Sphingolipid metabolism	14/583	47/6968	1.79E-05	0.00046
ko04976	Bile secretion	11/583	31/6968	2.29E-05	0.000523
ko00591	Linoleic acid metabolism	7/583	13/6968	3.04E-05	0.000624
ko00901	Indole alkaloid biosynthesis	7/583	14/6968	5.64E-05	0.001053
ko00950	Isoquinoline alkaloid biosynthesis	14/583	53/6968	7.87E-05	0.001347
ko00350	Tyrosine metabolism	21/583	103/6968	9.69E-05	0.00153
ko00945	Stilbenoid, diarylheptanoid and gingerol biosynthesis	8/583	20/6968	0.000116	0.001593
ko00965	Betalain biosynthesis	8/583	20/6968	0.000116	0.001593
ko04016	MAPK signaling pathway - plant	25/583	139/6968	0.000185	0.00237
ko00980	Metabolism of xenobiotics by cytochrome P450	11/583	40/6968	0.000312	0.003771
ko04726	Serotonergic synapse	7/583	19/6968	0.000571	0.006514
ko04712	Circadian rhythm - plant	12/583	53/6968	0.001151	0.012434
ko00941	Flavonoid biosynthesis	8/583	30/6968	0.002556	0.02623
ko04728	Dopaminergic synapse	11/583	52/6968	0.003248	0.031748
ko00590	Arachidonic acid metabolism	7/583	26/6968	0.004451	0.041532
ko04141	Protein processing in endoplasmic reticulum	26/583	184/6968	0.005386	0.048063

Table S3. Analysis of KOG function of HCD

Gene id	MeanTPM (Cd_HCD)	MeanTPM (CK_HCD)	log_2Fold-Change	KOG
Lminor_016999	30.51681	0.0001	18.21924	KOG0382-Carbonic anhydrase[R]
Lminor_010389	489.2249	160.725	1.605903	KOG0055-Multidrug/pheromone exporter, ABC superfamily[Q]
Lminor_015338	18.43099	6.392066	1.52778	KOG0054-Multidrug resistance-associated protein/mitoxantrone resistance protein, ABC superfamily[Q]
Lminor_013702	6.288235	2.597603	1.275474	KOG0054-Multidrug resistance-associated protein/mitoxantrone resistance protein, ABC superfamily[Q]
Lminor_014693	59.33858	28.31733	1.067285	K05666-ko02010 ABC transporters;ko04976 Bile secretion;ko01524 Platinum drug resistance
Lminor_002525	13.54982	36.27566	-1.42073	KOG0057-Mitochondrial Fe/S cluster exporter, ABC superfamily[U]
Lminor_008394	1.182684	3.831711	-1.69592	KOG0057-Mitochondrial Fe/S cluster exporter, ABC superfamily[U]
Lminor_017118	144.4931	51.1768	1.497439	KOG0102-Molecular chaperones mortalin/PBP74/GRP75, HSP70 superfamily[O]
Lminor_012904	13.19808	5.537933	1.252908	KOG1051-Chaperone HSP104 and related ATP-dependent Clp proteases[O]
Lminor_001367	28.35877	12.10072	1.228701	KOG0102-Molecular chaperones mortalin/PBP74/GRP75, HSP70 superfamily[O]
Lminor_003769	50.69138	23.10448	1.133568	KOG0356-Mitochondrial chaperonin, Cpn60/Hsp60p[O]
Lminor_008694	106.3543	50.29938	1.080266	KOG0020-Endoplasmic reticulum glucose-regulated protein (GRP94/endoplasmin), HSP90 family[O]
Lminor_007269	18.42877	8.857781	1.056943	KOG0546-HSP90 co-chaperone CPR7/Cyclophilin[O]
Lminor_012265	585.5768	4.129914	7.147603	KOG0710-Molecular chaperone (small heat-shock protein Hsp26/Hsp42)[O]
Lminor_012788	2.02055	0.231664	3.124643	KOG0101-Molecular chaperones HSP70/HSC70, HSP70 superfamily[O]
Lminor_016559	44.67185	7.505565	2.573334	KOG0100-Molecular chaperones GRP78/BiP/KAR2, HSP70 superfamily[O]

Lmi-nor_005296	160.2267	28.00536	2.51634	KOG0020-Endoplasmic reticulum glucose-regulated protein (GRP94/endoplasmin), HSP90 family[O]
Lmi-nor_018597	36.65982	8.971286	2.030813	KOG1051-Chaperone HSP104 and related ATP-dependent Clp proteases[O]
Lmi-nor_002559	22.63575	171.8644	-2.9246	KOG0207-Cation transport ATPase[P]

Table S4. Analysis of KOG function of HCD

Gene id	MeanTPM (Cd_LCD)	MeanTPM (CK_LCD)	log2Fold-Change	KOG
Lmi-nor_017138	1.916924	0.209895	3.191056	KOG0382-Carbonic anhydrase[R]
Lmi-nor_009468	54.84009	2.957491	4.212785	KOG0065-Pleiotropic drug resistance proteins (PDR1-15), ABC superfamily[Q]
Lmi-nor_004950	61.46276	4.875647	3.656047	KOG0065-Pleiotropic drug resistance proteins (PDR1-15), ABC superfamily[Q]
Lmi-nor_010389	691.2623	70.73606	3.288715	KOG0055-Multidrug/pheromone exporter, ABC superfamily[Q]
Lmi-nor_015338	25.7312	4.454855	2.530068	KOG0054-Multidrug resistance-associated protein/mitoxantrone resistance protein, ABC superfamily[Q]
Lmi-nor_001427	4.504249	1.516278	1.570752	KOG0059-Lipid exporter ABCA1 and related proteins, ABC superfamily[IR]
Lmi-nor_006278	13.29158	4.648158	1.515782	KOG0061-Transporter, ABC superfamily (Breast cancer resistance protein)[Q]
Lmi-nor_001426	1.573889	0.566352	1.474562	KOG0059-Lipid exporter ABCA1 and related proteins, ABC superfamily[IR]
Lmi-nor_010980	31.21878	14.76091	1.080633	KOG0064-Peroxisomal long-chain acyl-CoA transporter, ABC superfamily[I]
Lmi-nor_012265	96.92645	2.951634	5.037305	KOG0710-Molecular chaperone (small heat-shock protein Hsp26/Hsp42)[O]
Lmi-nor_016559	60.69791	9.435435	2.685486	KOG0100-Molecular chaperones GRP78/BiP/KAR2, HSP70 superfamily[O]
Lmi-nor_013606	26.79833	5.089339	2.396593	KOG0710-Molecular chaperone (small heat-shock protein Hsp26/Hsp42)[O]

Lminor_021169	33.59181	6.452953	2.380078	KOG0101-Molecular chaperones HSP70/HSC70, HSP70 superfamily[O]
Lminor_014170	256.7936	67.50997	1.927437	KOG0100-Molecular chaperones GRP78/BiP/KAR2, HSP70 superfamily[O]
Lminor_011658	150.3325	50.94483	1.561149	KOG1051-Chaperone HSP104 and related ATP-dependent Clp proteases[O]
Lminor_005296	32.88797	12.19523	1.431243	KOG0020-Endoplasmic reticulum glucose-regulated protein (GRP94/endoplasmin), HSP90 family[O]
Lminor_001318	10.20309	20.67786	-1.01908	KOG0356-Mitochondrial chaperonin, Cpn60/Hsp60p[O]
Lminor_001036	8.065104	2.529702	1.672726	KOG0207-Cation transport ATPase[P]
Lminor_006449	49.19964	13.03498	1.91626	KOG0207-Cation transport ATPase[P]

Disclaimer/Publisher's Note: The statements, opinions and data contained in all publications are solely those of the individual author(s) and contributor(s) and not of MDPI and/or the editor(s). MDPI and/or the editor(s) disclaim responsibility for any injury to people or property resulting from any ideas, methods, instructions or products referred to in the content.
